# Incidence and risk factors of glaucoma after surgery for congenital cataract diagnosed under one year of age: Protocol for Korean Nationwide Epidemiological Study for Childhood Glaucoma (KoNEC)

**DOI:** 10.1371/journal.pone.0264020

**Published:** 2022-02-17

**Authors:** Sooyeon Choe, Ahnul Ha, Sung Uk Baek, Jin-Soo Kim, Young Kook Kim

**Affiliations:** 1 Department of Ophthalmology, Seoul National University College of Medicine, Seoul, Korea; 2 Department of Ophthalmology, Seoul National University Hospital, Seoul, Korea; 3 Department of Ophthalmology, Jeju National University Hospital, Jeju-si, Korea; 4 Department of Ophthalmology, Hallym University College of Medicine, Chuncheon, Korea; 5 Department of Ophthalmology, Hallym University Sacred Heart Hospital, Anyang, Korea; 6 Department of Ophthalmology, Chungnam National University Sejong Hospital, Sejong, Korea; Cairo University Kasr Alainy Faculty of Medicine, EGYPT

## Abstract

**Introduction:**

Congenital cataract (CC) can cause childhood visual impairment, even after CC surgery, due to subsequent occurrence of glaucoma. The post-CC-surgery glaucoma study results vary, due largely to the lack of a sufficient number of population-based cohort studies. This study herein proposed aims to assess the incidence and risk factors of post-CC-surgery glaucoma in a nationwide cohort. The clinico-demographic factors associated with outcomes of post-CC-surgery glaucoma will be investigated as well.

**Materials and methods:**

This population-based, nested case-control study is planned as part of the Korean Nationwide Epidemiological Study for Childhood Glaucoma (KoNEC). Data for a nationwide retrospective cohort representative of the years 2008 to 2018 will be extracted from the National Institutes of Health database, which includes demographic information, diagnoses and medical visits as well as procedures, records of prescriptions, and comorbidities. Among the patients whose first CC diagnosis was made before age 1, only those who underwent surgery for CC will be included in the study. The rate of occurrence of post-CC-surgery glaucoma will be determined based on a Poisson distribution. Also, for cumulative incidence plotting, the Kaplan-Meier method will be used. To identify risk factors for occurrence and poor outcomes of post-CC-surgery glaucoma, we will perform a multivariable regression analysis of matched samples. The detailed patterns of post-CC-surgery glaucoma management will be studied as well.

**OSF registration number:**

DOI 10.17605/OSF.IO/AWTEC.

## Introduction

Congenital cataract (CC) is one of the most common diseases causing childhood blindness. Nowadays, CC surgeries are performed to prevent blindness. However, risk of blindness remains even after treatment of CC. In this regard, postoperative glaucoma is a potential threat of visual impairment [1]. The previous reports show post-CC-surgery glaucoma occurrence rates ranging widely from 5.3 to 58.7%, due mainly to differences in study-population characteristics and follow-up durations [2–11].

For treatment of post-CC-surgery glaucoma, antiglaucoma eyedrops are administered; however, some patients, for whom medication is inadequate, have to undergo additional glaucoma surgery [12–14]. As the long-term outcomes are often revealed to be poor, efforts have been made to identify risk factors. Although discordances among those studies exist, they have indicated possible associations of post-CC-surgery glaucoma development with age at detection of cataract and at cataract surgery, primary intraocular lens implantation, microphthalmia, and usage of trypan blue [2, 3, 5, 7, 15–18].

However, those reports share a limitation, which is a small database due to the relatively low incidence of post-CC-surgery glaucoma. Additionally, the factors affecting the outcome of post-CC-surgery glaucoma have not been well identified. Herein we present our plan for a nationwide study to determine the incidence and clinico-demographic factors associated with post-CC-surgery glaucoma in Korean children. Also, we will identify factors contributing to post-CC-surgery glaucoma outcomes and perform a descriptive analysis of post-CC-surgery glaucoma management.

## Materials and methods

### Study design and setting

This study will be a population-based, nested case-control study utilizing national health claims from 2008 to 2018 as collected by the Health Insurance Review and Assessment (HIRA) service of South Korea.

This study is part of the Korean Nationwide Epidemiological Study for Childhood Glaucoma (KoNEC), which plans for a nationwide epidemiological study of both primary glaucoma (i.e., primary congenital glaucoma, juvenile open-angle glaucoma) and secondary glaucoma (i.e., Sturge-Weber syndrome-associated glaucoma, steroid-induced glaucoma, postoperative glaucoma). We have registered this study’s protocol in the Open Science Framework (OSF; registration number, DOI 10.17605/OSF.IO/AWTEC).

### Data sources and data collection

The HIRA database (based on National Institutes of Health (NIH) data), is a repository of claims submitted for healthcare provider reimbursement. Since South Korea has a system of universal coverage, HIRA includes almost 50 million patients representing 98% of the total population [19]. The HIRA Deliberative Committee approved, for the purposes of this study, conditional extraction of data, based on the following understanding: that all information personally identifiable would be masked, and that Resident Registration Numbers would be replaced with an identification key randomly assigned for each patient. To determine the total age-less-than-1-year population of South Korea, Population and Housing Census data (Korean Statistical Information Service; available at: http://kosis.kr) will be used.

HIRA holds medical-claim-related data on demographics, diagnoses, medical appointments, procedures, prescriptions, and comorbidities for the entire population of South Korea [20, 21]. The diagnoses therein are coded from the Korean Standard Classification of Diseases, 7th Revision, which is the International Classification of Diseases, 10th Revision, amended to fit the Korean situation. The potential risk factors for post-CC-surgery glaucoma that can be accessed based on the HIRA database will be obtained. Demographic factors, medical institution factors, procedural factors, and ophthalmic anomalies (congenital malformations of anterior segment, microphthalmia, persistent fetal vasculature, congenital malformation of retina) are all possible risk factors for post-CC-surgery glaucoma (Table 1). Also, to determine if any systemic diseases are associated with increased risk of post-CC-surgery glaucoma, the following will be included in the analysis: 1) genetic and metabolic comorbidities (Down syndrome, Hallermann-Streiff syndrome, Lowe syndrome, galactosemia, Marfan syndrome, trisomy 13–15, neonatal hypoglycemia, Alport syndrome, myotonic dystrophy, Fabry disease, hypoparathyroidism, Conradi-Hunermann syndrome, incontinentia pigmenti) and 2) infectious history (Toxoplasmosis, Coxackievirus, Syphilis, Varicella-Zoster, HIV, Parvo B19, Rubella, Cytomegalovirus, Herpes Simplex [HSV-1, HSV-2]) (Table 1).

**Table 1:** Summary of included possible risk factors for post-congenital cataract-surgery glaucoma.

Classifications	Characteristics
Demographics	Age at diagnosis
	Age at procedure
	Sex
Medical institution	Type of hospital by level of health care (tertiary, general hospital, clinic)
	Location (city, rural)
Procedures	Number of cataract surgeries (unilateral, bilateral)
	Type of cataract surgery (aphakia, primary IOL implantation, secondary IOL implantation)
	Time from diagnosis to cataract surgery
Genetic & Metabolic comorbidities	Down syndrome
	Hallermann-Streiff syndrome
	Lowe syndrome
	Galactosemia
	Marfan syndrome
	Trisomy 13–15
	Neonatal hypoglycemia
	Alport syndrome
	Myotonic dystrophy
	Fabry disease
	Hypoparathyroidism
	Conradi-Hunermann syndrome
	Incontinentia pigmenti
Infections	Toxoplasmosis
	Coxackievirus
	Syphilis
	Varicella-Zoster
	HIV
	Parvo B19
	Rubella
	Cytomegalovirus
	Herpes Simplex (HSV-1, HSV-2)
Ophthalmic anomalies	Congenital malformations of anterior segment
	Microphthalmia
	Persistent fetal vasculature
	Congenital malformation of retina

HIV = Human Immunodeficiency Virus, HSV = Herpes Simplex Virus, IOL = intraocular lens.

Additionally, the information on antiglaucoma agents (prostaglandin analogue, beta blockers, adrenergic agonists, carbonic anhydrase inhibitors) and glaucoma surgery (trabeculectomy and Ahmed glaucoma valve implantation) will be obtained to identify management patterns of post-CC-surgery glaucoma.

### Study subjects

**Fig 1:**
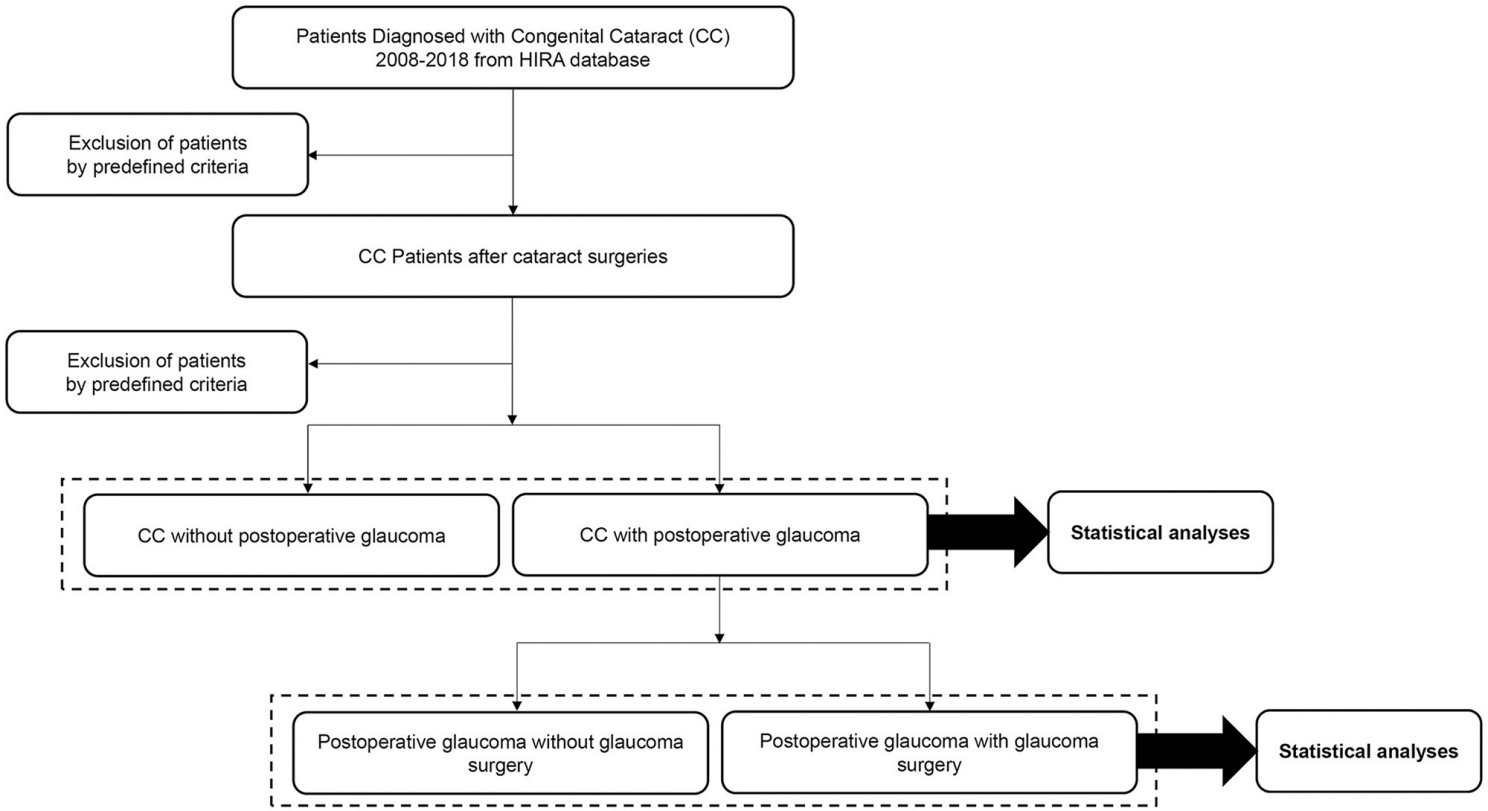
Flowchart of study subjects.

All patients diagnosed with CC between 2008 and 2018, in HIRA, will be included as candidates. Among them, those having undergone surgery for CC will be included. Excluded will be: (1) patients diagnosed with CC prior to 2008, and (2) those first diagnosed with CC after age 1. The remaining subjects will be designated as CC patients after cataract surgeries. From them, the following patients will be excluded: (1) those with a follow-up period of less than 1 year after CC surgery, (2) those diagnosed with glaucoma prior to CC surgery, and (3) those prescribed an antiglaucoma agent prior to CC surgery. Among them, patients having used an antiglaucoma agent for longer than 3 months will be grouped as patients with post-CC-surgery glaucoma, and the others will be grouped as patients without post-CC-surgery glaucoma (Fig 1). We will investigate the occurrence rate and the risk factors of post-CC-surgery glaucoma. Also, for the patients with post-CC-surgery glaucoma, we plan to further analyze the probability of glaucoma surgery. The post-CC-surgery glaucoma cases in which glaucoma surgery had been necessary despite usage of anti-glaucoma eye drops will be defined as “poor outcome.” The factors associated with poor outcome of post-CC-surgery glaucoma also will be investigated.

### Statistical analysis plan

A descriptive analysis of all of the possible risk factors for post-CC-surgery glaucoma will be performed. The continuous variables will be presented with either the mean and standard deviation or the median and interquartile values, according to the data distribution. The categorical variables will be reported in frequency tables (n, %).

The post-CC-surgery glaucoma occurrence rates will be calculated as the number of patients having post-CC-surgery glaucoma divided by the number of patients having undergone CC surgery. Male-to-female ratios for the annual incidence rates will also be calculated. The incidence rates along with the 95% confidence intervals (CIs) of post-CC-surgery glaucoma will be estimated per 1,000 person-years, based on a Poisson distribution. The Kaplan-Meier method will be applied for a survival analysis of the probability curves of the development of post-CC-surgery glaucoma and glaucoma surgery.

A case-control study will be nested into the CC patient cohort. These cases are members of the cohort who had developed post-CC-surgery glaucoma. For each case, up to three randomly selected controls from the cohort were matched for age at the time of first CC surgery. With these matched case controls, a multivariable analysis of the risk factors will be performed by logistic regression. The possible risk factors included in this study are listed in Table 1. The level of statistical significance will be set as 2-sided P < 0.05. All of the analyses will be performed with SAS Enterprise Guide version 7.1 (SAS Inc., Cary, NC, USA).

### Ethics and declaration

Approvals for data collection and analysis have been obtained prospectively from the Korean Health Authorities (M20200519540, approved on February 10, 2021) and the Institutional Review Board/Ethics Committee of Seoul National University Hospital (E-2011-103-1173, approved on November 23, 2020). The data will be managed confidentially according to national security law and its guidelines. The study will adhere to the principles of the Declaration of Helsinki.

### Status of study

We received specific approval for use of the NIH database regarding rare diseases based on its data-usage guidelines on February 10, 2021, and we are currently in the process of preparing for data extraction.

## Discussion

Our study will be a nationwide, population-based cohort study investigating the occurrence rate and risk factors for post-CC-surgery glaucoma as well as the poor-outcome factors of post-CC-surgery glaucoma. Whereas previously reported occurrence rates of post-CC-surgery glaucoma differ widely, this study will provide results derived from a single, huge national-population database. Notably, ours will be the first study to report nationwide post-CC-surgery glaucoma data for an Asian ethnicity. Also, it will prove novel in reporting management and poor-outcome risk factors of post-CC-surgery glaucoma on a nationwide basis.

### Strengths

Since the incidences of post-CC-surgery glaucoma naturally are low, a large cohort study is essential for obtainment of representative results. The HIRA database stores all of the medical information covered by the universal health insurance system of South Korea. As the surgical treatment of CC is well established, there will be little chance of erroneous inclusion of incorrect diagnoses in the database. Thus, the cohort that we obtained from the HIRA database will certainly include almost all patients diagnosed with CC between 2008 and 2018. And since our database covers 11 years, our study can be expected to reveal long-term clinical outcomes of post-CC-surgery glaucoma. A further strength of our proposed study, which will be particularly valuable considering the difficulties commonly encountered in the treatment of post-CC-surgery glaucoma, is that it will offer tips for better management of such patients.

Multivariable models of risk factors will be analyzed under a predefined analysis plan in the study protocol. Indeed, via the HIRA database, information including a large number of possible risk factors can be easily aggregated to obtain extensive analysis outcomes.

### Limitations

This study is retrospective in nature. Although prospective studies afford high-level evidence, answering research questions in cases of rare diseases renders prospective studies infeasible. Particularly as information on post-CC-surgery glaucoma is limited, retrospective studies are necessary. This study will use a large, nationwide database and a predefined protocol to minimize the incurring of methodological bias from its retrospective design.

The HIRA database we procured for this study is constituted of medical claims made through the NHI system. Therefore, information not included in medical claims, such as visual acuity, intraocular pressure, gonioscopic appearance, specific surgical procedures and socioeconomic status, are unobtainable. We will investigate possible risk factors other than those for which information is unavailable. And since the HIRA database represents the nation of South Korea, the proposed study will include an all-Korean cohort; thus there will be no accounting for ethnic differences in this study. Note also that cases in which operations were performed outside of Korea will not be included in the HIRA database, and thus will not be considered in the study. Finally, as we will include cases diagnosed with CC under one year of age to exclude juvenile cataract, CC cases with delayed presentation to health-care facilities might be missed.

## Conclusion

Epidemiological characteristics of post-CC-surgery glaucoma in Korean children will be investigated. The study’s established protocol will prevent selection bias and improve the reproducibility and transparency of the research.

### Dissemination plans

We will disseminate research findings through publication of peer-reviewed papers and/or presentations of study findings at academic conferences. Amendments to and termination of the study will be reported to the Institutional Review Board/Ethics Committee of Seoul National University Hospital.

## Abbreviations

CC: congenital cataract
HIRA: Health Insurance Review and Assessment
NIH: National Institutes of Health
